# CRISPR Explorer: A fast and intuitive tool for designing guide RNA for genome editing

**DOI:** 10.14440/jbm.2016.138

**Published:** 2016-10-10

**Authors:** Kenian Chen, Yi Jin, Yin C. Lin

**Affiliations:** Baylor Institute for Immunology Research, Dallas, TX 75246, USA

**Keywords:** CRISPR-Cas9, guide RNA design, open-source web application

## Abstract

The RNA-guided CRISPR-Cas9 (clustered, regularly interspaced, short palindromic repeat-CRISPR-associated 9) system has become a revolutionary technology for targeted genome engineering. The critical step of this technology requires the design of a highly specific and efficient guide RNA (gRNA) that will guide the Cas9 nuclease to the complementary DNA target sequence. CRISPR-Explorer is a new and user-friendly web server for selecting optimal CRISPR sites. It implements the latest scoring schemes of gRNA specificity and efficiency based on published empirical studies. The gRNA design results are generated instantly, thus removing wait times. The user can visualize the high-quality gRNAs with detailed design information through an interactive genome browser. Furthermore, the user can define and specify the parameters for gRNA selection in the Batch Design mode, which recognizes various input formats. CRISPR Explorer is freely accessible at: http://crisprexplorer.org.

## HIGHLIGHT

CRISPR-Explorer facilitates the design of high-quality gRNA for the low- and high-throughput needs of researchers due to its ease of use, comprehensiveness, and instantaneous feedback. The advantages of our web application over other existing web applications are: (1) fast: gRNA design results based on published empirical studies are generated instantly, thus removing wait times; (2) intuitive: the user can visualize the high-quality gRNAs with detailed design information through an interactive genome browser; and (3) flexible: the user can define his/her own parameters for gRNA selection and various input formats are recognized. The scoring schemes that are used include the specificity score and the efficiency score. Targeting multiple regions can be achieved simultaneously through Batch Design. Guide RNA design results can be ranked and filtered on the browser before export. The user will select the genome of interest for genome editing, the desired gRNA length, and the cognate promoter (U6 or T7) that drives the gRNA expression. After selecting these options, the user will have the choice to input a gene name (official gene symbol, Ensembl ID, or Refseq ID), genome coordinates, or a DNA sequence for gRNA visualization on the CRISPR-Explorer browser. In the Browse mode, potential gRNA candidates are color-coded based on their specificities and efficiencies to facilitate selection, and detailed information for an individual gRNA design can be displayed in a pop-up. In the Batch mode, the user can input a gene, a gene list, a genome coordinate, or a list of genome coordinates. The output of the “Batch Design Exporter” is an interactive table that allows the user to rank and/or filter his/her gRNA designs. The table can be exported in a text file format.

## INTRODUCTION

CRISPR-Cas is an RNA-guided nuclease system that allows efficient perturbation of gene functions and is increasingly being used for genome-wide functional screens [1-3]. The type II CRISPR-Cas system from *Streptococcus pyogenes* Cas9 (SpCas9) has been adapted to target a specific DNA sequence for Cas9 nuclease cleavage using a programmable guide RNA (gRNA) [4-7]. Targeting is mediated by the first 20 nucleotides at the 5’-end of the gRNA, which are complementary to the target DNA sequence, followed by a 3 bp protospacer adjacent motif (PAM) sequence. Cas9 nuclease can be directed by a programmable gRNA to induce DNA double-stranded breaks (DSBs) at specific locations in the genome. In mammalian cells, genome editing typically occurs through the repair of the Cas9-induced DSB by the error-prone non-homologous end-joining (NHEJ) mechanism, which introduces variable-length insertion/deletion (indel) mutations, or through homology-directed (HDR) repair in the presence of an exogenous DNA template. An indel mutation at the spliced coding exon of the target gene frequently results in a coding frameshift and the initiation of nonsense-mediated decay of the gene transcript, which causes gene inactivation. In addition to its utility in genome editing, by removing the Cas9 nuclease activity, catalytically inactive Cas9 (dCas9) proteins that are fused to a co-activation or co-repression domain can be guided by gRNA to a specific DNA sequence to activate or repress gene transcription, respectively [8].

Numerous studies have shown that CRISPR-Cas9 not only targets its intended on-target sites, but also certain off-target sites in the genome that share sequence similarity with the on-target sites [9-12]. This off-target effect is attributed to the ability of Cas9 to recognize a non-canonical PAM sequence and tolerate the nucleotide mismatch between gRNA and its target sequence. To achieve productive genome editing, the efficiency of Cas9-mediated modification is also critical. The sequence model predicts that the editing efficiency is influenced by the DNA target sequence as well as the flanking sequences [13]. Indeed, such a sequence model has been experimentally validated, and gRNA efficiency can be calculated based on this model [13]. Therefore, to generate a highly specific and efficient gRNA design, we believe that it is important to consider both the specificity and efficiency of the gRNA.

To date, a number of web-based applications have been developed to select highly specific genome-editing sites [9, 12, 14, 15]. Typically, these applications require the target sequence or gRNA as input. The potential CRISPR sites on the target sequence are identified, and this is followed by the searching of potential off-target sites throughout the reference genome. The wait time depends on the computation time for each target site calculation. The wait time increases as the number of input sequences increases, which can result in a long wait time. The output is often presented on a plain web page that provides a basic display of the positions of the CRISPR sites that match the input sequence. A few applications use the UCSC Genome Browser to display results that are not interactive. Most of these applications only consider the specificity of the gRNA, but not the efficiency. One application, CRISPR-ERA, does consider the specificity and efficiency for gRNA design but uses an ad hoc scoring scheme [14]. Finally, most of the existing web applications lack an intuitive and convenient way for browsing and batch design.

Here, our goal was to develop a guide RNA design web server that eliminates the above issues and offers the most up-to-date and proven scoring schemes for gRNA specificity and efficiency. CRISPR-Explorer is fast, intuitive, and flexible. It is particularly useful for genome-wide functional screening and high-throughput screening using the CRISPR-Cas9 technology. The CRISPR-Cas system is a rapidly moving technology. We will continue to update the scoring schemes as new knowledge and data become available, and we expect to incorporate the scoring schemes for new Cas nucleases that are identified in the future [16].

Briefly, the whole reference genomes of human (hg19) and mouse (mm10) for every possible CRISPR site (*e.g.* 5’(N~20)-NGG, 5’(N~20)-NAG sites, etc.) were scanned. To identify potential off-targets for each possible gRNA, we used the “all-mapper” of the Genome Multitool (GEM) mapper, which gives all alignments of a specific short sequence with a user-defined number of mismatches [17]. The number of off-target alignments in the reference genome for a specific CRISPR site grows rapidly as the number of mismatches increases, and therefore, up to 4 mismatches in the 20 bp gRNA were allowed when searching for off-target sites. Three mismatches were used for the truncated (18 bp) gRNA since previous studies showed that a truncated gRNA with more than 3 mismatches usually does not have a detectable off-target effect [18]. To calculate the specificity score (or aggregate scores of single hits), we adopted the algorithm that was developed by the Zhang F group [12] and incorporated their experimentally determined effect on targeting of each mismatch position (*M*= [0, 0, 0.014, 0, 0, 0.395, 0.317, 0, 0.389, 0.079, 0.445, 0.508, 0.613, 0.851, 0.732, 0.828, 0.615, 0.804, 0.685, 0.583]). As for the calculation of the truncated (18 bp) gRNA, the first two nucleotide positions and their experimentally determined effects were removed from the calculation of the specificity score. The efficiency score was calculated using the SSC program [13].

To achieve fast query speed, the results were sorted and indexed using the Tabix program [19]. We adopted the WashU Epigenome Browser [20] as the presentation framework for display customization and linking to the gRNA database. The batch design exporter that we implemented is based on the Angular UI Grid (http://ui-grid.info/) JavaScript framework, which provides an interactive output.

## PROCEDURE

### Input

**Figure 1:**
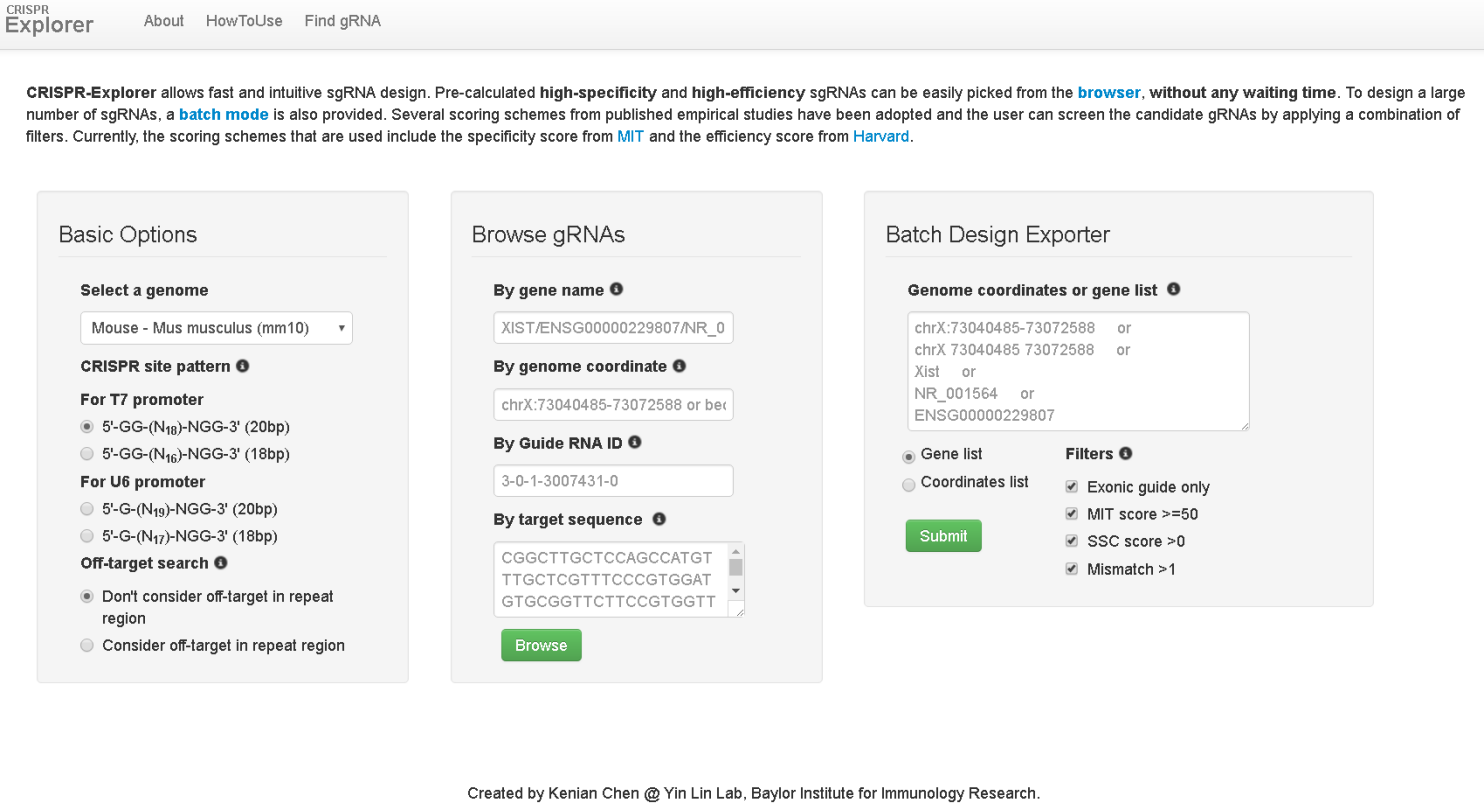
A screen shot of the CRISPR-Explorer homepage that shows the three input panels: “Basic Options”, “Browse gRNAs”, and “Batch Design Exporter”.

The CRISPR-Explorer homepage contains 3 panels. See **Figure 1**.

1. ‘Basic Options’ panel. Choose the genome that you want to edit and which promoter you are planning to use. Currently, only Cas9 PAM (-NGG) is supported because it is the most widely used and has a more mature scoring scheme based on empirical studies. However, we will include other PAMs (such as Cpf1) in the future when possible. By default, repeat regions are excluded for off-target searching. However, the user has the option to include repeat regions during the gRNA design.
2. ‘Browse gRNAs’ panel. Browse gRNAs for your gene of interest by entering the gene name, the genome coordinate, or a DNA sequence from the genome. The input format for the gene name can be the official gene symbol, Ensembl gene name, or refGene name. If you know the genomic location of the region, you can directly query the browser with the genome coordinate (either in bed format or the following format: chrX:NNNNN-NNNNN). When the target sequence (with a limit of 20000 bases) is supplied as input, the Blat program (an alignment tool) from the UCSC Genome Browser is used to map the sequence to the genome location. The speed of this part depends on the UCSC Blat server. A guide RNA ID that is generated from Batch Design Exporter can be used to access the detailed information about the gRNA.
3. ‘Batch Design Exporter’ panel. Multiple genome coordinates or gene entries can be entered simultaneously. Currently, a maximum number of 100 entries is allowed per run to prevent server overload. You can also reduce the output and get a faster response by applying pre-defined filters. The ‘Exonic guide only’ option selects gRNAs that fall within the exonic regions. RefGene provides the gene structure to define the exonic regions. The ‘MIT score ≥ 50’ option selects gRNAs that have specificity scores greater than or equal to 50 [12]. The ‘SSC score > 0’ option selects gRNAs that have efficiency scores that are greater than zero [13]. The ‘Mismatch > 1’ option selects for gRNAs that have more than 1 mismatch with the most similar sequence in the whole genome.

### Output

**Figure 2.**
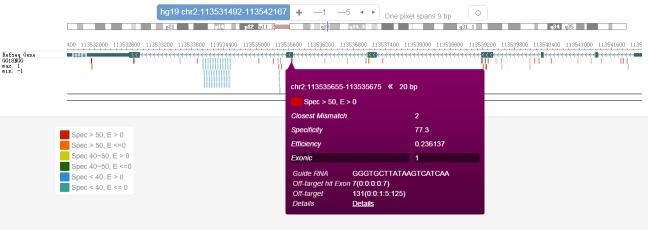
**An example of the browser track display of “Browse gRNA”.** The human IL1A gene was used as an input. By clicking on the gRNA, the pop-up box reveals detailed information about it. The “Details” link directs the user to off-target sequence information.

**Figure 3.**
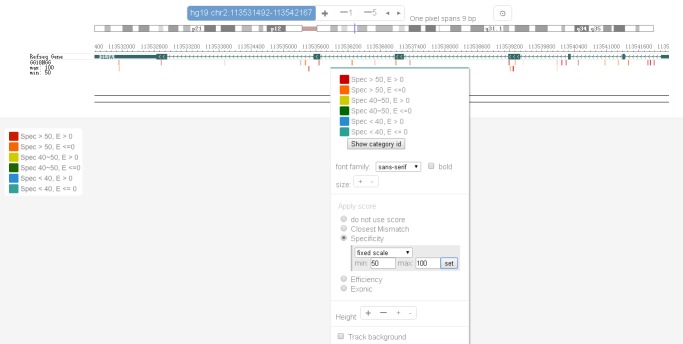
**An example of the browser track display of “Browse gRNA”.** The human IL1A gene was used as an input. Selecting “Configure” after right clicking on the track allows the user to choose the gRNAs that are displayed based on closest mismatch, specificity and efficiency scores.

**Figure 4.**
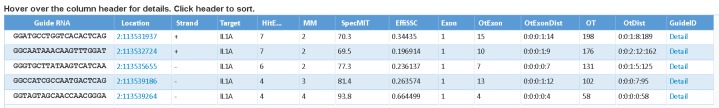
**The interactive table of the *IL1A* gene.** The filters of “Exonic guide only”, “MIT score ≥ 50”, “SSC score > 0”, and “Mismatch > 1” are applied before the generation of this table.

The two main display options in CRISPR-Explorer are the interactive browser (**Fig. 2** and **3**) and a table (**Fig. 4**).

4. After the ‘Browse’ button inside the ‘Browse gRNAs’ panel is clicked, the browser launches (**Fig. 2**). The gRNAs are color-coded based on different specificity and efficiency score cutoffs. The legend on the bottom left-hand side defines the color codes. Red gRNAs have the highest specificity and efficiency scores, and green gRNAs have the lowest specificity and efficiency scores. By rolling the cursor over the gRNA bar, brief information about that gRNA is displayed. To get detailed information about a gRNA, clicking on the gRNA leads to a pop-up window that shows the gRNA’s genome coordinate, strand-specificity, length, closest mismatch, specificity and efficiency scores, sequence, and detailed off-target information (#OT) (**Fig. 2**). A click on the ‘Details’ link in this pop-up will lead to a page with detailed information about the off-target sequences of the selected gRNA. The table that is found on this page is interactive and contains the following information: off-target sequences with the mismatches in upper case letters (‘Off-target sequence’), genome coordinate with strand specificity that is indicated by “+” or “-” (‘Location’), number of mismatches (‘Mismatch’), off-target score (‘Score’), and the name of the gene in which the exons are hit by the off-target mismatch (‘Hit Exon’).
5. To change the gRNA display on the browser, first right-click on the browser track and then click on ‘Configure’. One can choose different filters (‘Closes Mismatch’, ‘Specificity’, or ‘Efficiency’) to limit the gRNAs that are displayed on the browser (**Fig. 3**). For example, in **Figure 3**, the selection of ‘Specificity’ with the scoring range between 50 and 100 restricts the display of gRNAs to those whose scores fall within this range.
6. After the ‘Submit’ button inside the ‘Batch Design Exporter’ panel is clicked, an interactive table launches (**Fig. 4**). When a gene name is used as input, the output will contain information about the exon (ranked in composite gene model) in which the target site falls (‘HitExon’ column). If a transcript name is use as input (*e.g.* Ensembl transcript name), the ‘HitExon’ will rank by that transcript. Genome coordinates is the ideal input format for gRNA design in unannotated genomic regions. When genome coordinates are supplied as input, the ‘HitExon’ information is not reported.
7. The interactive table generated by the ‘Batch Design Exporter’ contains the following information: gRNA sequence (‘Guide RNA’), genome coordinates (‘Location’), strand specificity (‘Strand’), target’s name (‘Target’), exon number where the gRNA falls (‘HitExon’), number of mismatches (‘MM’), specificity score (‘SpecMIT’), efficiency score (‘EffiSSC’), exonic or not (‘Exon’), number of potential off-targets that is exonic (‘OtExon’), number of mismatches contained by the potential off-targets that fall in exons (‘OtExonDist’), number of potential off-targets in the whole genome (‘OT’), number of mismatches contained by the potential off-targets that fall in the genome (‘OtDist’), and the link to the detailed information about the off-target sequences (‘GuideID’) (**Fig. 4**). The description in the ‘OtExonDist’ column (for example, 0:0:0:0:4 in **Fig. 4**, first row) means that all of the predicted targets have 4 mismatches. In this example, none of the predicted off-targets have zero, one, two or three mismatches.
8. Each column of the interactive table can be ranked alphabetically or numerically from lowest to highest or vice versa. This can be achieved by clicking on the right of the individual header of the first row of the table where an up or down arrow is.
9. Different features can be selected for the ‘Guide RNA’, ‘Location’, ‘Strand’, ‘Target’, ‘Exon’, ‘OtExon’, ‘OtExonDist’, ‘OT’, and ‘OtDist’ columns. The filtering functions (equals/less/or greater) are available for the ‘HitExon’, ‘MM’, ‘SpecMIT’, and ‘EffiSSC’ columns. This can be achieved by rolling the cursor to the left of the individual column header. Then, click on the ‘Ξ’ icon that appears and the filtering option will be revealed.
10. After filtering and ranking, the personally customized table can be exported as a ‘.csv’ file by clicking the “Export to CSV” button at the bottom of the table. The user can keep the default file name or type in the file name inside the textbox that is above the “Export to CSV” button.

## TROUBLESHOOTING/DEBUGGING

Please contact the Webmaster, Kenian.Chen@BSWHealth.org, for any requests, comments, and bug reporting. We expect to maintain this web application for at least two years and may provide updates that include the newly identified CRISPR systems that target RNA.

## Abbreviations used

gRNA: guide RNA
PAM: protospacer adjacent motif
DSBs: double-stranded breaks
NHEJ: non-homologous end-joining
HDR: homology-directed

## References

[1] Shalem O., Sanjana N. E., Hartenian E., Shi X., Scott D. A. (2013). Genomescale CRISPR-Cas9 knockout screening in human cells. Science.

[2] Wang T., Wei J. J., Sabatini D. M., Lander E. S. (2014). Genetic screens in human cells using the CRISPR-Cas9 system. Science.

[3] Zhou Y., Zhu S., Cai C., Yuan P., Li C. (2014). High-throughput screening of a CRISPR/Cas9 library for functional genomics in human cells. Nature.

[4] Deltcheva E., Chylinski K., Sharma C. M., Gonzales K., Chao Y. (2011). CRISPR RNA maturation by trans-encoded small RNA and host factor RNase III. Nature.

[5] Garneau J. E., Dupuis M., Villion M., Romero D. A., Barrangou R. (2010). The CRISPR/Cas bacterial immune system cleaves bacteriophage and plasmid DNA. Nature.

[6] Jinek M., Chylinski K., Fonfara I., Hauer M., Doudna J. A. (2012). A programmable dual-RNA-guided DNA endonuclease in adaptive bacterial immunity. Science.

[7] Sapranauskas R., Gasiunas G., Fremaux C., Barrangou R., Horvath P. (2011). The Streptococcus thermophilus CRISPR/Cas system provides immunity in Escherichia coli. Nucleic Acids Res.

[8] Gilbert L. A., Larson M. H., Morsut L., Liu Z., Brar G. A. (2013). CRISPRmediated modular RNA-guided regulation of transcription in eukaryotes. Cell.

[9] Bae S., Park J., Kim J. (2014). Cas-OFFinder: a fast and versatile algorithm that searches for potential off-target sites of Cas9 RNA-guided endonucleases. Bioinformatics.

[10] Cho S. W., Kim S., Kim J. M., Kim J. (2013). Targeted genome engineering in human cells with the Cas9 RNA-guided endonuclease. Nat Biotechnol.

[11] Fu Y., Foden J. A., Khayter C., Maeder M. L., Reyon D. (2013). High-frequency off-target mutagenesis induced by CRISPR-Cas nucleases in human cells. Nat Biotechnol.

[12] Hsu P. D., Scott D. A., Weinstein J. A., Ran F. A., Konermann S. (2013). DNA targeting specificity of RNA-guided Cas9 nucleases. Nat Biotechnol.

[13] Xu H., Xiao T., Chen C., Li W., Meyer C. A. (2015). Sequence determinants of improved CRISPR sgRNA design. Genome Res.

[14] Liu H., Wei Z., Dominguez A., Li Y., Wang X. (2015). CRISPR-ERA: a comprehensive design tool for CRISPR-mediated gene editing, repression and activation. Bioinformatics.

[15] Moreno-Mateos M. A., Vejnar C. E., Beaudoin J., Fernandez J. P., Mis E. K. (2015). CRISPRscan: designing highly efficient sgRNAs for CRISPR-Cas9 targeting in vivo. Nat Methods.

[16] Slaymaker I. M., Gao L., Zetsche B., Scott D. A., Yan W. X. (2015). Rationally engineered Cas9 nucleases with improved specificity. Science.

[17] Marco-Sola S., Sammeth M., Guigó R., Ribeca P. (2012). The GEM mapper: fast, accurate and versatile alignment by filtration. Nat Methods.

[18] Fu Y., Sander J. D., Reyon D., Cascio V. M., Joung J. K. (2014). Improving CRISPR-Cas nuclease specificity using truncated guide RNAs. Nat Biotechnol.

[19] Li H. (2011). Tabix: fast retrieval of sequence features from generic TABdelimited files. Bioinformatics.

[20] Zhou X., Maricque B., Xie M., Li D., Sundaram V. (2011). The Human Epigenome Browser at Washington University. Nat Methods.

